# The Impact of Spirituality, Religiosity, and Quality of Life on the Survival of Patients with Head-and-Neck Squamous Cell Carcinoma in Brazil: A Cohort Study

**DOI:** 10.1007/s10943-026-02655-w

**Published:** 2026-04-16

**Authors:** Agna Soares da Silva Menezes, Cristina Paixão Durães, Larissa Lopes Fonseca, Tatiana Almeida de Magalhães, Jairo Evangelista Nascimento, Gracielle Soares da Silva Ruas, Antonio Alvimar de Souza, Arlen de Paulo Santiago Filho, Adriana Aparecida Almeida de Aguiar Ribeiro, Alfredo Maurício Batista de Paula, Sérgio Henrique Sousa Santos, Lucyana Conceição Farias, André Luiz Sena Guimarães

**Affiliations:** 1 Department of Dentistry, Universidade Estadual de Montes Claros (Unimontes), Montes Claros, Minas Gerais, Brazil; 2 Department of Philosophy, Universidade Estadual de Montes Claros (Unimontes), Montes Claros, Minas Gerais, Brazil; 3 Dilson Godinho Hospital, Montes Claros, Minas Gerais, Brazil; 4 Institute of Agricultural Sciences, Universidade Federal de Minas Gerais (UFMG), Montes Claros, Minas Gerais, Brazil; 5 Laboratório de Pesquisa Em Saúde, Hospital Universitário Clemente Faria, Universidade Estadual de Montes Claros, 562 Av. Cula Mangabeira Santo Expedito, Montes Claros, MG 39401-001 Brazil

**Keywords:** Cancer, Quality of life, Spirituality, Religiosity, Religious-spiritual coping

## Abstract

This prospective cohort study investigated whether spirituality, religiosity, emotional symptoms, and quality of life were associated with survival in patients with head-and-neck squamous cell carcinoma (HNSCC) treated at a tertiary referral center in Brazil. A total of 245 adults were enrolled at baseline, including 118 patients with HNSCC and 127 companions without a history of cancer. Only the 118 HNSCC patients were included in the survival analyses. Participants were evaluated using validated instruments for religiosity/spirituality, depression, anxiety, and quality of life. Survival time was calculated in days from baseline assessment to death or censoring, and associations with psychosocial variables were examined using bivariate comparisons, complementary correlation analyses, and Kaplan–Meier survival curves. During follow-up, patients who died had significantly higher depressive symptom scores and lower organizational religiosity than survivors. Longer survival was positively associated with organizational religiosity and self-rated quality of life, whereas depressive symptoms were inversely associated with survival duration. These findings suggest that greater organizational religiosity and better self-perceived quality of life may be associated with longer survival in patients with HNSCC. In contrast, depressive symptoms may be associated with poorer survival outcomes.

**Supplementary Information:**

The online version contains supplementary material available at 10.1007/s10943-026-02655-w.

## Introduction

Cancer is the leading cause of death and a significant barrier to increasing life expectancy in virtually every country worldwide (Sung et al., 2021). The global cancer mortality rate averages 5.55 per 100,000 individuals and is notably higher in men (9.95 per 100,000) (Sung et al., 2021). The rising global burden of cancer incidence and mortality is primarily attributed to population aging, demographic expansion, and shifts in the prevalence of significant risk factors, many of which are linked to socioeconomic conditions (Sung et al., 2021). Among malignancies, HNSCC ranks as the fifth most prevalent cancer worldwide, accounting for approximately 780,000 new diagnoses annually, and thus constitutes a pressing global public health challenge (De Paula et al., 2009; Sung et al., 2021).

HNSCC predominantly affects men and is strongly associated with behavioral risk factors such as tobacco use and excessive alcohol consumption. Human papillomavirus (HPV) infection has also emerged as a significant etiological factor (Fonseca–Silva et al., 2012; Marques–Silva et al., 2012). Radiotherapy (RT) remains a cornerstone of HNSCC treatment; however, it often results in adverse effects that impair patients’ quality of life (QOL) (Ortigara et al., 2021).

QOL is a multidimensional construct encompassing physical, psychological, environmental, and social dimensions (Samsoe et al., 2022). As cancer diagnosis and treatment can be deeply distressing, numerous studies have highlighted the protective influence of spiritual and religious experiences in promoting QOL and psychological resilience (Werdani, 2022). These experiences have been shown to provide a sense of hope, purpose, and emotional support during oncological illness.

Defining spirituality and religiosity within the health sciences presents challenges due to the lack of universally accepted definitions (Cook, 2004). Spirituality is often described as a personal pursuit of meaning, purpose, and connection with the transcendent or sacred. In contrast, religion typically encompasses structured beliefs, practices, and rituals oriented toward a higher power, often practiced within communal settings (Koenig, 2009; Koenig et al., 1997). Religiosity can be classified as organizational (e.g., attending religious services) or non-organizational (e.g., personal prayer or reading spiritual texts) (Goncalves et al., 2015; Puchalski et al., 2004). Religion is widely recognized as a crucial coping mechanism in the context of chronic illness, providing emotional comfort, helping maintain self-esteem, and fostering long-term psychological adjustment (Koenig, 2009; Pahlevan Sharif et al., 2021). In the oncology setting, religious faith is frequently used to manage cancer-related stress (Goncalves et al., 2015; Sharif et al., 2018). When religion is used as a method for managing health-related stress, this is referred to as religious/spiritual coping. This coping can be positive, leading to feelings of hope, connection, and comfort, or negative, when it contributes to feelings of abandonment, low self-worth, or existential despair (Pargament et al., 2000; Seeman et al., 2003).

Despite medical advances, a cancer diagnosis often evokes fear, existential questioning, and a confrontation with mortality. In this context, religiosity and spirituality can play a dual role: they may help reduce emotional suffering while contributing to improvements in QOL (Leal et al., 2022). Spirituality and religiosity may be present before the cancer diagnosis or may intensify as a result of the disease, functioning as a source of inner strength, cultural guidance, and psychological support (Jim et al., 2015). Studies have consistently demonstrated that spirituality and religiosity positively influence cancer patients’ quality of life (QoL) (Goncalves et al., 2015; Seeman et al., 2003). Therefore, QoL may mediate between psychosocial factors and clinical outcomes (Fleck et al., 2000).

The present study aims to evaluate the associations between religious/spiritual coping, religiosity, QoL, and survival in patients with HNSCC undergoing oncological treatment. We hypothesize that patients with higher levels of organizational religiosity and better QoL, particularly in the physical and psychological domains, will have improved survival outcomes.

## Methods

### Ethical Approval

All procedures involving human subjects were conducted in accordance with the ethical standards of institutional and national research committees, the 1964 Declaration of Helsinki and its subsequent amendments, or comparable ethical standards. The Research Ethics Committee approved this study under no Certificate of Presentation for Ethical Review (CAAE): 12031119.8.0000.5146. All survey participants agreed to sign the informed consent form.

### Study Design

The study was conducted at the outpatient referral clinic for cancer patients at Dilson de Quadros Godinho Hospital in Montes Claros, MG, Brazil. This longitudinal cohort study took place from May 2019 to May 2021. The study followed the STROBE guidelines for cohort reporting (von Elm et al., 2008). Recruitment, exposure, follow-up, and data collection occurred throughout this period.

### Population and Sampling

To determine the sample size for this study, we aimed to achieve a statistical power of 95% with an alpha level of 0.05. We considered the incidence of oral cancer in Brazil, which accounts for approximately 5% of new cancer cases, along with historical data from the health service on expected new patient numbers. Consequently, we aimed to recruit at least 112 patients. Eligible patients were recruited consecutively. Specifically, all patients who met the inclusion criteria and attended the oncology outpatient clinic during the study period were invited to participate. This consecutive recruitment strategy was adopted to minimize selection bias and improve the representativeness of the clinical sample.

We included individuals with a histological diagnosis of HNSCC affecting the tongue, gingiva, floor of the mouth, palate, tonsil, oropharynx, pyriform sinus, and hypopharynx. Participants must be 18 or older and have signed an informed consent form. We excluded individuals with cognitive impairments, speech difficulties, neurological diseases that hindered the interview process, or diagnoses other than HNSCC.

To provide contextual reference values for spirituality, religiosity, and psychosocial variables among adults without a cancer diagnosis, we invited the companions accompanying patients to the outpatient clinic (aged ≥ 18 years and with no self-reported history of cancer) to participate in the baseline assessment. This pragmatic approach, evaluating patients together with informal companions or caregivers from the same sociocultural environment, is widely used in psychosocial oncology, where patient–companion dyads offer meaningful contrasts for interpreting coping behaviors, emotional responses, and spiritual constructs in serious illness contexts (Delgado–Guay et al., 2013; Koenig & Carey, 2025; Taylor & Mamier, 2005).

This strategy helps reduce confounding arising from cultural, socioeconomic, and environmental differences that would be expected if unrelated community controls were used. Importantly, the companions were not intended to be statistically representative of the broader Brazilian adult population; instead, they served as a socioculturally matched non-cancer reference group, allowing us to differentiate pre-existing spiritual and emotional characteristics from those potentially shaped by the cancer diagnosis. Their participation was limited to the baseline phase, as the sole purpose was to establish unbiased contextual reference values. All participants provided written informed consent.

### Data collection and Instruments

The patients were approached during the interval between chemotherapy and radiotherapy sessions. After receiving detailed information about the study and signing the informed consent form, participants completed individual, private interviews. To guide these interviews, we applied a structured set of validated instruments: a socioeconomic and clinical questionnaire, the religious/spiritual coping (RCOPE) scale, the Duke religious index (DUREL), the WHOQOL-SRPB (Spirituality, Religiosity, and Personal Beliefs Module), the WHOQOL-Bref, the Beck depression inventory (BDI), and the Beck anxiety inventory (BAI). Mortality data for survival analysis were collected through the mortality information system (MIS) database.

The selection and application of spiritual and religious measurement instruments in this study were guided by best practice recommendations for validation and cross-cultural use (Koenig & Al Zaben, 2021) and aligned with recent methodological guidance emphasizing the avoidance of scale contamination between spirituality and indicators of mental or physical health (Koenig & Carey, 2025).

The RCOPE scale (Panzini & Bandeira, 2005), adapted and validated for the Brazilian population, includes 87 items assessing religious coping responses in stressful health contexts. For this study, we used the eight positive religious/spiritual coping subscales (e.g., seeking spiritual growth and transformation of self/life) and the four negative coping subscales (e.g., negative reappraisal of meaning and dissatisfaction with God), which are most relevant for evaluating adaptive and maladaptive coping mechanisms in the context of cancer. According to the original validation study, these subscales demonstrated high internal consistency: Cronbach’s alpha was 0.92 for positive coping and 0.88 for negative coping (Panzini & Bandeira, 2005).

The WHOQOL-Bref was used to assess health-related quality of life across four domains: physical, psychological, social, and environmental. This 26-item instrument is widely validated and appropriate for use in oncologic populations. In the Brazilian validation study, internal consistency values for the domains ranged from α = 0.72 to 0.86 (Fleck et al., 2000).

The WHOQOL-SRPB, a spirituality module developed to complement the WHOQOL instruments, was also fully applied, encompassing eight facets: spiritual connection, meaning in life, awe, wholeness and integration, spiritual strength, inner peace, hope and optimism, and faith. These items address culturally and existentially relevant aspects of spirituality and belief systems. The Brazilian adaptation of the SRPB demonstrated excellent internal consistency: Cronbach’s alpha = 0.94 (Krageloh et al., 2015; Panzini et al., 2011).

The BDI (Beck et al., 1961) and the BAI (Beck et al., 1988) were applied in full, each comprising 21 items evaluating depressive and anxiety symptoms, respectively. These instruments have been validated in Brazilian populations and have shown strong internal reliability: BDI α = 0.89 and BAI α = 0.91 (Gorenstein & Andrade, 1996).

The Duke religion index (DUREL) (Koenig et al., 1997) was used to assess three dimensions of religiosity: organizational, non-organizational, and intrinsic religiosity. However, the internal consistency of the DUREL in our sample (Cronbach’s α = 0.621) is slightly lower than values reported in some Brazilian validations (Lucchetti et al., 2012). Psychometric reviews emphasize that Cronbach’s alpha must be interpreted in light of the number of items, sample characteristics, and scale dimensionality. Short instruments, particularly those with fewer than 10 items, commonly present lower alpha values, even when conceptually robust (Tavakol & Dennick, 2011). Additional methodological reviews (Taber, 2017) note that while α ≥ 0.70 is often recommended, coefficients around 0.60 may still be acceptable in exploratory studies, clinical samples, or heterogeneous populations, provided that the scale is theoretically coherent and demonstrates evidence of validity. In this context, the Cronbach’s α of 0.621 observed in our sample falls within acceptable ranges for brief instruments and aligns with expectations for five-item religiosity measures such as the DUREL.

### Statistical Analysis

All statistical analyses were performed using IBM SPSS Statistics version 28.0 (IBM Corp., Armonk, NY, USA), configured in Brazilian Portuguese. The distribution of continuous variables was assessed using the Kolmogorov–Smirnov test. Normally distributed variables were compared using one-way ANOVA with Tukey’s post hoc tests, whereas non-normally distributed variables were analyzed with the Kruskal–Wallis and Mann–Whitney U tests. Categorical variables were examined using Chi-square or Fisher’s exact tests, as appropriate.

Before inferential analyses, internal consistency coefficients were recalculated for all psychometric instruments to ensure reliability within the present sample. Cronbach’s alpha values indicated strong internal consistency for WHOQOL-SRPB (α = 0.944), WHOQOL-Bref (α = 0.766), CRE/RCOPE (α = 0.936), BDI (α = 0.846), and BAI (α = 0.880). As expected for brief multidimensional scales, the five-item DUREL showed moderate reliability (α = 0.621). Complete reliability outputs generated by SPSS (Portuguese interface) are provided in the Supplementary Material.

Survival analysis was performed to investigate associations between psychosocial constructs such as religious/spiritual coping, religiosity, and quality of life and overall survival in patients with HNSCC. The event of interest was death due to HNSCC or treatment-related complications, verified through the Brazilian mortality information system (MIS). The time origin (T₀) was defined as the date of histopathological diagnosis. Patients alive at 24 months were right-censored. Non-informative censoring was assumed, in accordance with recommended methodological standards (Dey et al., 2020). Survival probabilities were estimated using the Kaplan–Meier (KM) method, and differences between groups were assessed using the log-rank test.

As requested during the peer-review process, all Kaplan–Meier curves, including those corresponding to non-significant variables, were generated and are provided in full as supplementary SPSS output files (Portuguese interface), ensuring complete transparency of the survival analyses. To evaluate independent predictors of survival while adjusting for covariates, Cox proportional hazards (PH) regression models were applied. All candidate variables were initially included in a multivariable model, and backward elimination was used to retain predictors with statistical significance (*p* < 0.05). The proportional hazards assumption was rigorously evaluated. For categorical predictors, log-minus-log survival plots were inspected for parallel patterns over time. For continuous predictors, time-dependent covariates (covariate × time interaction terms) were incorporated to test for violations statistically. No significant departures from the PH assumption were detected, confirming the suitability of the final Cox model. All analyses adhered to established guidelines for survival analysis in epidemiological and clinical research (Dey et al., 2020).

## Results

**Fig. 1:**
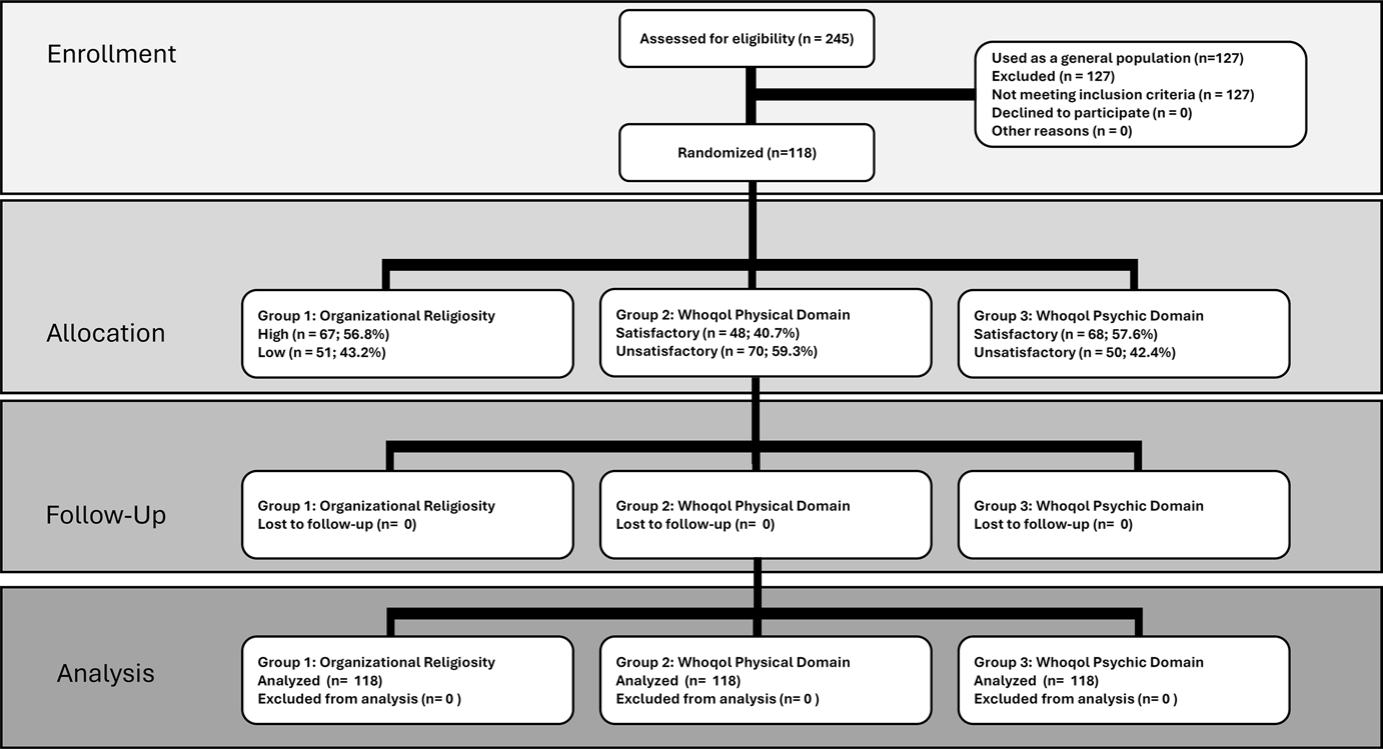
*Study flowchart*. A total of 245 participants were included in the study, consisting of 118 patients diagnosed with head-and-neck squamous cell carcinoma (HNSCC). Initially, 127 companions were analyzed to assess potential bias but were subsequently excluded from the follow-up phase

A total of 245 participants were enrolled at baseline, including 118 patients diagnosed with head-and-neck squamous cell carcinoma (HNSCC) and 127 companions without a history of cancer. Companions were included only at baseline to assess potential sociodemographic and psychosocial comparability and were not included in the longitudinal survival analyses. Eligible HNSCC patients were recruited consecutively during routine attendance at the oncology outpatient clinic. Patients were followed for at least 24 months after completion of oncologic treatment to assess survival outcomes (Fig. 1).

Baseline comparisons between patients and companions are presented in Table 1. Statistically significant differences were observed between groups for sex (*p* < 0.001), religion (*p* = 0.046), and monthly income (*p* = 0.030), whereas marital status (*p* = 0.151), anxiety symptoms assessed by the Beck anxiety inventory (BAI; *p* = 0.443), and depressive symptoms assessed by the Beck depression inventory (BDI; *p* = 0.547) did not differ significantly between groups. In both groups, most participants reported catholic affiliation, having a partner, and a monthly income of up to two minimum wages.

**Table 1:** Sociodemographic characteristics and mental health indicators among patients with head-and-neck squamous cell carcinoma (HNSCC) and their companions at baseline (*N* = 245)

Variable	Companions (*n* = 127)	HNSCC patients (*n* = 118)	*p* value
*Sex*			0.000*
Male	43 (31.9%)	92 (68.1%)	
Female	84 (76.4%)	26 (23.6%)	
*Religion*			0.046*
Catholic	94 (48.5%)	100 (51.5%)	
Evangelical	26 (66.7%)	13 (33.3%)	
None	7 (58.3%)	5 (41.7%)	
*Marital status*			0.151
With partner	65 (52.9%)	58 (47.1%)	
Without partner	62 (50.8%)	60 (49.2%)	
*Income*			0.030*
≤ 1 minimum wage	64 (44.1%)	81 (55.9%)	
> 1 minimum wage	63 (63.0%)	37 (37.0%)	
*Beck Anxiety Inventory (BAI)*			0.443
No symptoms	70 (54.3%)	59 (45.7%)	
Mild anxiety	24 (47.1%)	27 (52.9%)	
Moderate anxiety	19 (43.2%)	25 (56.8%)	
Severe anxiety	14 (66.7%)	7 (33.3%)	
*Beck Depression Inventory (BDI)*			0.547
No symptoms	73 (53.7%)	63 (46.3%)	
Mild depression	16 (40.0%)	24 (60.0%)	
Moderate depression	21 (51.2%)	20 (48.8%)	
Severe depression	17 (60.7%)	11 (39.3%)	

HNSCC, head-and-neck squamous cell carcinoma; *p* values were calculated using Pearson’s Chi-square test or Fisher’s exact test, as appropriate. Percentages are presented by category (row percentages). *p < 0.05

Among the 118 HNSCC patients included in the cohort, the survival analyses focused exclusively on this group. Internal consistency coefficients for the psychometric instruments used in the present sample were recalculated and are provided in the Supplementary Material. Bivariate comparisons between patients who died during follow-up and those who survived are shown in Table 2. Patients who died had significantly higher depressive symptom scores than survivors (BDI: 12.06 ± 5.30 vs. 8.56 ± 5.84; t = 3.03; df = 124; *p* = 0.003). In addition, survivors presented significantly higher organizational religiosity scores than deceased patients (DUREL-RO: 4.11 ± 1.41 vs. 3.45 ± 1.80; t = −2.12; df = 123; *p* = 0.036). Anxiety scores did not differ significantly between groups (BAI: 7.06 ± 7.44 vs. 6.51 ± 7.37; *t* = 0.37; df = 124; *p* = 0.711). General quality of life scores were numerically higher among survivors, although this difference did not reach statistical significance in the direct group comparison (WHOQOL overall score: 66.26 ± 14.95 vs. 60.61 ± 17.15; *t* = −1.80; df = 124; *p* = 0.075).

**Table 2:** Psychosocial Variables and Survival Outcomes

Variable	Deceased (Mean ± SD)	Survivors (Mean ± SD)	*t*	df	*p* value	Significance
BDI Score	12.06 ± 5.30	8.56 ± 5.84	3.03	124	0.003	**
BAI Score	7.06 ± 7.44	6.51 ± 7.37	0.37	124	0.711	ns
Organizational Religiosity (DUREL_RO)	3.45 ± 1.80	4.11 ± 1.41	− 2.12	123	0.036	*
General QoL (WHOQOL)	60.61 ± 17.15	66.26 ± 14.95	− 1.8	124	0.075	ns

Comparison of psychosocial variables between patients who survived and those who died during follow-up. *t*-tests were used for continuous variables. Results include means, standard deviations, t-values, degrees of freedom, and *p* values. ns, not significant. *p < 0.05, **p < 0.01

**Fig. 2:**
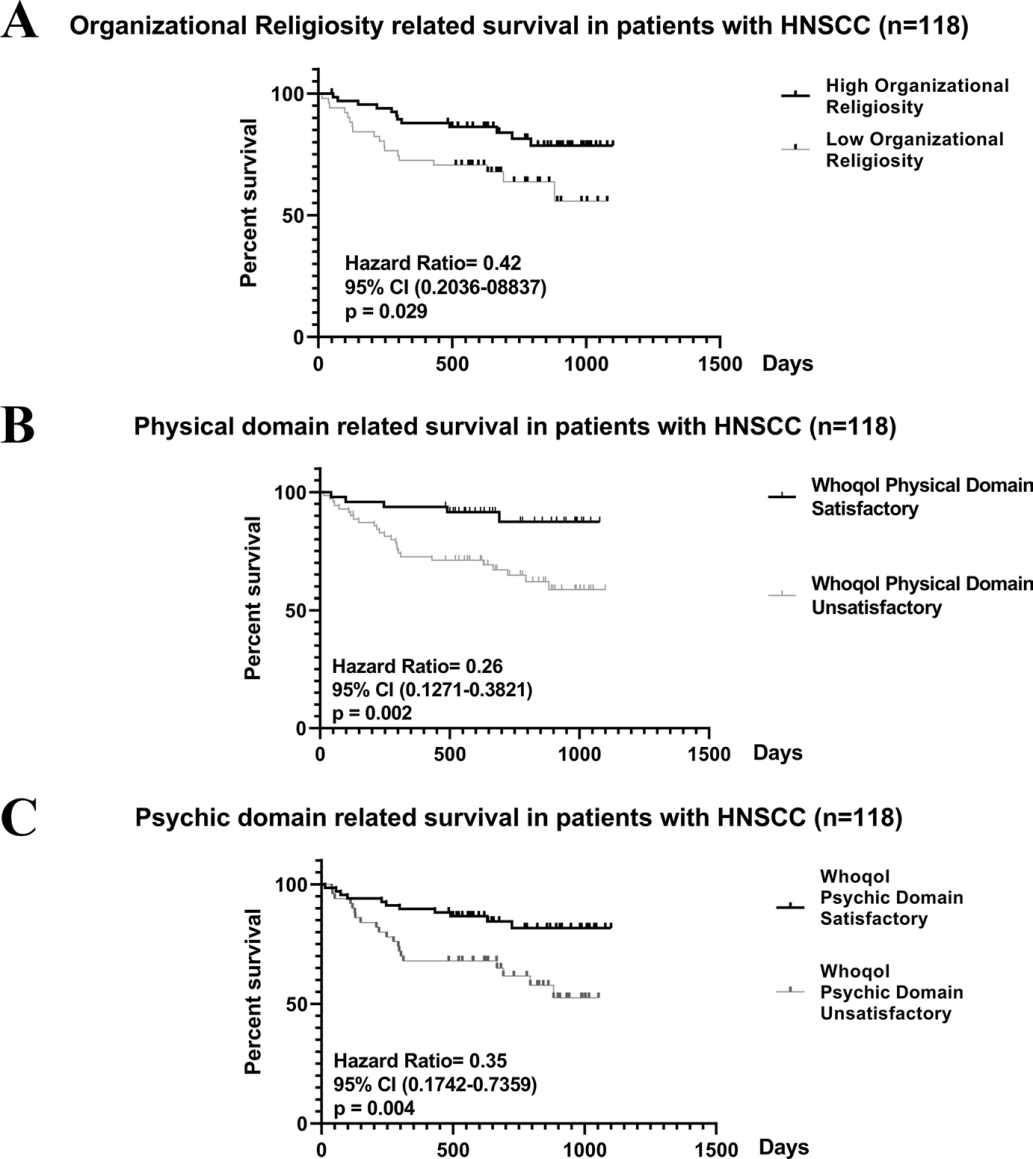
*Significant factors related to HNSCC patient survival.* In panel A, patients with higher organizational religiosity presented higher survival rates than those with lower religiosity. In panel B, according to the World Health Organization Quality of Life (WHOQOL) scale, patients with higher scores in the physical domain also experienced increased survival. In panel C, patients with better psychological domain scores showed more prolonged survival

Kaplan–Meier survival analyses further demonstrated that patients with higher organizational religiosity showed better survival probabilities over time. Similarly, higher scores in the physical and psychological domains of quality of life were associated with longer survival, as illustrated in Fig. 2. These findings indicate that, within this cohort, organizational religiosity and specific dimensions of quality of life were positively associated with survival. In contrast, greater depressive symptom burden was associated with poorer survival outcomes.

## Discussion

The clinical and sociodemographic profile of patients with HNSCC observed in our study, predominantly male, over 50 years old, with low income and education, aligns with well-established epidemiological patterns (De Paula et al., 2009; Pinheiro et al., 2014). HNSCC remains a highly aggressive malignancy with frequent cervical metastasis and poor prognosis, especially in socioeconomically vulnerable populations (De Paula et al., 2009). In Brazil, the majority of cases (76%) are diagnosed at advanced stages (III and IV), reflecting systemic delays in diagnosis and access to care (De Paula et al., 2009). Our sample mirrored this reality: Only 12.6% of patients were diagnosed at stages I or II, while 87.4% presented with advanced-stage disease, thereby limiting the feasibility of staging-based survival comparisons. Importantly, no significant differences were observed between HNSCC patients and their companions in terms of anxiety and depression, supporting previous findings in similar populations (Fernandes Ferreira et al., 2020). This strengthens the methodological choice to include companions as a control reference group. This enables a more accurate attribution of spiritual and emotional differences to the cancer experience rather than baseline disparities.

Our findings also demonstrated a positive association between more prolonged survival and higher levels of organizational religiosity (OR), consistent with previous studies. For example, the National Health Interview Survey reported that 69% of cancer patients pray for health purposes, compared to 45% in the general US population (Ross et al., 2008). Religiosity and spirituality have been shown to buffer the effects of stress on physiological systems, including cardiovascular reactivity, inflammation, and the hypothalamic–pituitary–adrenal axis, all of which may influence disease outcomes (Patterson et al., 2018; Seeman et al., 2003). Emotional benefits derived from religiosity/spirituality (R/E), such as greater coping capacity and social support, may reinforce health-promoting behaviors (Aldwin et al., 2014; Koenig, 2009; Puchalski et al., 2004).

Conversely, spiritual distress or perceived abandonment by a religious community is linked to worse psychological outcomes and lower adherence to medical care among cancer patients (Jim et al., 2015; Koenig, 2009; Park et al., 2009; Sherman et al., 2005). R/E frameworks may thus function as a form of emotional and behavioral self-regulation that enhances patient resilience (Goncalves et al., 2015; Puchalski et al., 2004). Studies have reported significant correlations between higher religiosity, better quality of life (QOL), and prolonged survival (Chida et al., 2009; Goncalves et al., 2015). Additionally, reframing the cancer experience as a form of spiritual growth, even if symbolic, may help patients maintain an emotional connection and a sense of purpose despite adversity (Rudaz et al., 2018). Although few spiritually based interventions have been systematically tested in oncology populations, the available evidence suggests that they may improve QOL and survival (Goncalves et al., 2015).

In the present study, patients with more prolonged survival (in days) had better QOL in the physical and psychological domains. Data from the literature show that QOL is an important prognostic factor for cancer patients since low scores are associated with a worse prognosis (Bonanno et al., 2007; Siddiqui et al., 2008). A previous study on the association between QOL and survival in cancer patients showed that patients with high QOL scores had a statistically significantly higher survival rate than those with low QOL scores (Ganz et al., 1991; Ruckdeschel & Piantadosi, 1994). The shock of the diagnosis, the pain and stress of the treatment, physical and intellectual restrictions, daily activity limitations, social stigmatization, and dealing with situations that put the patient’s life at risk must be considered (Puchalski et al., 2004). Thus, in addition to the classical evaluation, assessing the impact of the disease on QOL is essential as a valuable tool for instituting additional treatment, rehabilitation, palliative care, and psychological and social interventions aimed at the patient’s integral well-being (Detmar et al., 2002). Data from the literature show that QOL is an important prognostic factor in cancer patients (Ruckdeschel & Piantadosi, 1994; Siddiqui et al., 2008).

This study presents several limitations that should be considered. First, it was conducted at a single referral center, which may limit the generalizability of the findings to broader or more diverse populations. Second, although the inclusion of companions at baseline provided socio-culturally matched non-cancer reference values, this group does not reflect the broader Brazilian adult population and therefore cannot serve as a probabilistic community control. Their role was exclusively contextual, strengthening internal comparisons but reducing external validity. Third, the observational design precludes causal inference, and residual confounding cannot be ruled out entirely.

The psychometric evaluation of the instruments in our sample confirmed strong reliability for all major scales, including WHOQOL-SRPB, WHOQOL-Bref, CRE/RCOPE, BDI, and BAI. As expected for brief multidimensional measures, the DUREL presented a moderate internal consistency (*α* = 0.621) (Taber, 2017; Tavakol & Dennick, 2011). Prior psychometric literature indicates that Cronbach’s alpha tends to be lower in short scales or in instruments assessing heterogeneous content domains, without necessarily compromising validity or interpretability (Tavakol & Dennick, 2011). Nonetheless, this moderate reliability may have contributed to restricted variance in religiosity scores, and this factor should be taken into account when interpreting the associations observed.

In conclusion, patients with higher physical and psychological quality of life and greater organizational religiosity exhibited significantly longer survival. These findings underscore the importance of integrating quality-of-life assessments and spiritual support strategies into comprehensive care for individuals with head-and-neck squamous cell carcinoma. Future multicenter and longitudinal studies are needed to validate these associations, examine the evolution of spiritual coping and psychosocial well-being throughout the cancer trajectory, and determine whether structured psychosocial or spiritual interventions can positively influence survival and clinical outcomes.

## Supplementary Information

Below is the link to the electronic supplementary material.

Supplementary file1 (PDF 218 kb)

## Declarations

### Standards of Reporting

The study was reported according to STROBE for cohort reporting guidelines (von Elm et al., 2008).
