# Supplementary material for: The Impact of Spirituality, Religiosity, and Quality of Life on the Survival of Patients with Head-and-Neck Squamous Cell Carcinoma in Brazil: A Cohort Study
Source: J Relig Health. 2026 Apr 16;65(4):3601–15. doi: 10.1007/s10943-026-02655-w (PMC13481469; doi:10.1007/s10943-026-02655-w)
Supplement: Supplementary file 1 [file 10943_2026_2655_MOESM1_ESM.pdf]

Appendix – Study Instruments (English Version)

WHOQOL-SRPB (32 items)

The WHOQOL-SRPB assesses: Spiritual Connection, Meaning and Purpose in Life, Experiences of Awe, Wholeness & Integration, Spiritual Strength, Inner Peace, Hope & Optimism, and Faith. Item reproduction is restricted by WHO copyright; only domain descriptions are provided.

Appendix – WHOQOL-SRPB (32 items)

Each item is rated on a 5-point Likert scale as follows:

- 1 = Not at all
- 2 = A little
- 3 = A moderate amount
- 4 = Very much
- 5 = An extreme amount

1. SP1.1 To what extent does any connection to a spiritual being help you to get through hard times?

    - 1 = Not at all
    - 2 = A little
    - 3 = A moderate amount
    - 4 = Very much
    - 5 = An extreme amount

2. SP1.2 To what extent does any connection to a spiritual being help you to tolerate stress?

    - 1 = Not at all
    - 2 = A little
    - 3 = A moderate amount
    - 4 = Very much
    - 5 = An extreme amount

3. SP1.3 To what extent does any connection to a spiritual being help you to understand others?

- 1 = Not at all
- 2 = A little
- 3 = A moderate amount
- 4 = Very much
- 5 = An extreme amount

4. SP1.4 To what extent does any connection to a spiritual being provide you with comfort / reassurance?

   - 1 = Not at all
   - 2 = A little
   - 3 = A moderate amount
   - 4 = Very much
   - 5 = An extreme amount

5. SP2.1 To what extent do you find meaning in life?

   - 1 = Not at all
   - 2 = A little
   - 3 = A moderate amount
   - 4 = Very much
   - 5 = An extreme amount

6. SP2.2 To what extent does taking care of other people provide meaning of life for you?

   - 1 = Not at all
   - 2 = A little
   - 3 = A moderate amount
   - 4 = Very much
   - 5 = An extreme amount

7. SP2.3 To what extent do you feel your life has a purpose?

   - 1 = Not at all
   - 2 = A little
   - 3 = A moderate amount
   - 4 = Very much
   - 5 = An extreme amount

8. SP2.4 To what extent do you feel you are here for a reason?

   - 1 = Not at all
   - 2 = A little
   - 3 = A moderate amount
   - 4 = Very much
   - 5 = An extreme amount

9. SP3.1 To what extent are you able to experience awe from your surroundings?

   - 1 = Not at all
   - 2 = A little
   - 3 = A moderate amount
   - 4 = Very much
   - 5 = An extreme amount

10. SP3.2 To what extent do you feel spiritually touched by beauty?

    - 1 = Not at all
    - 2 = A little
    - 3 = A moderate amount
    - 4 = Very much
    - 5 = An extreme amount

11. SP3.3 To what extent do you have feelings of inspiration / excitement in your life?

    - 1 = Not at all
    - 2 = A little
    - 3 = A moderate amount
    - 4 = Very much
    - 5 = An extreme amount

12. SP3.4 To what extent are you grateful for the things in nature that you can enjoy?

    - 1 = Not at all
    - 2 = A little
    - 3 = A moderate amount

- 4 = Very much
- 5 = An extreme amount

13. SP4.1 To what extent do you feel any connection between your mind, body and soul?

- 1 = Not at all
- 2 = A little
- 3 = A moderate amount
- 4 = Very much
- 5 = An extreme amount

14. SP4.2 How satisfied are you that you have a balance between mind, body and soul?

- 1 = Not at all
- 2 = A little
- 3 = A moderate amount
- 4 = Very much
- 5 = An extreme amount

15. SP4.3 To what extent do you feel the way you live is consistent with what you feel and think?

- 1 = Not at all
- 2 = A little
- 3 = A moderate amount
- 4 = Very much
- 5 = An extreme amount

16. SP4.4 How much do your beliefs help you to create coherence between what you do, think and feel?

- 1 = Not at all
- 2 = A little
- 3 = A moderate amount
- 4 = Very much
- 5 = An extreme amount

17. SP5.1 To what extent do you feel inner spiritual strength?

- 1 = Not at all
- 2 = A little
- 3 = A moderate amount
- 4 = Very much
- 5 = An extreme amount

18. SP5.2 To what extent can you find spiritual strength in difficult times?

- 1 = Not at all
- 2 = A little
- 3 = A moderate amount
- 4 = Very much
- 5 = An extreme amount

19. SP5.3 How much does spiritual strength help you to live better?

- 1 = Not at all
- 2 = A little
- 3 = A moderate amount
- 4 = Very much
- 5 = An extreme amount

20. SP5.4 To what extent does your spiritual strength help you to feel happy in life?

- 1 = Not at all
- 2 = A little
- 3 = A moderate amount
- 4 = Very much
- 5 = An extreme amount

21. SP6.1 To what extent do you feel peaceful within yourself?

- 1 = Not at all
- 2 = A little
- 3 = A moderate amount
- 4 = Very much
- 5 = An extreme amount

22. SP6.2 To what extent do you have inner peace?

- 1 = Not at all
- 2 = A little
- 3 = A moderate amount
- 4 = Very much
- 5 = An extreme amount

23. SP6.3 How much are you able to feel peaceful when you need to?

- 1 = Not at all
- 2 = A little
- 3 = A moderate amount
- 4 = Very much
- 5 = An extreme amount

24. SP6.4 To what extent do you feel a sense of harmony in your life?

- 1 = Not at all
- 2 = A little
- 3 = A moderate amount
- 4 = Very much
- 5 = An extreme amount

25. SP7.1 How hopeful do you feel?

- 1 = Not at all
- 2 = A little
- 3 = A moderate amount
- 4 = Very much
- 5 = An extreme amount

26. SP7.2 To what extent are you hopeful about your life?

- 1 = Not at all
- 2 = A little
- 3 = A moderate amount

- 4 = Very much
- 5 = An extreme amount

27. SP7.3 To what extent does being optimistic improve your quality of life?

- 1 = Not at all
- 2 = A little
- 3 = A moderate amount
- 4 = Very much
- 5 = An extreme amount

28. SP7.4 How able are you to remain optimistic in times of uncertainty?

- 1 = Not at all
- 2 = A little
- 3 = A moderate amount
- 4 = Very much
- 5 = An extreme amount

29. SP8.1 To what extent does faith contribute to your well-being?

- 1 = Not at all
- 2 = A little
- 3 = A moderate amount
- 4 = Very much
- 5 = An extreme amount

30. SP8.2 To what extent does faith give you comfort in daily life?

- 1 = Not at all
- 2 = A little
- 3 = A moderate amount
- 4 = Very much
- 5 = An extreme amount

31. SP8.3 To what extent does faith give you strength in daily life?

- 1 = Not at all
- 2 = A little
- 3 = A moderate amount
- 4 = Very much
- 5 = An extreme amount

32. SP8.4 To what extent does faith help you to enjoy life?

- 1 = Not at all
- 2 = A little
- 3 = A moderate amount
- 4 = Very much
- 5 = An extreme amount

WHOQOL-BREF (26 items)

Measures four domains: Physical Health, Psychological Health, Social Relationships, and Environment. Full item text is copyright-restricted; therefore, domain descriptions only are included.

Appendix B. WHOQOL-BREF (English Version)

The following questions ask about how you feel about your quality of life, health, and other areas of your life.

Please answer all questions thinking about the last two weeks. Choose the response that best represents your situation.

1. Quality of life (G1)

How would you rate your quality of life?

1 = Very poor | 2 = Poor | 3 = Neither poor nor good | 4 = Good | 5 = Very good

2. Satisfaction with health (G4)

How satisfied are you with your health?

1 = Very dissatisfied | 2 = Dissatisfied | 3 = Neither satisfied nor dissatisfied | 4 = Satisfied | 5 = Very satisfied

3. Pain and discomfort (F1.4)

To what extent do you feel that (physical) pain prevents you from doing what you need to do?

1 = Not at all | 2 = A little | 3 = A moderate amount | 4 = Very much | 5 = An extreme amount

4. Dependence on medication (F11.3)

How much do you need medical treatment to function in your daily life?

1 = Not at all | 2 = A little | 3 = A moderate amount | 4 = Very much | 5 = An extreme amount

5. Positive feelings (F4.1)

How much do you enjoy life?

1 = Not at all | 2 = A little | 3 = A moderate amount | 4 = Very much | 5 = An extreme amount

6. Meaning in life (F24.2)

To what extent do you feel your life to be meaningful?

1 = Not at all | 2 = A little | 3 = A moderate amount | 4 = Very much | 5 = An extreme amount

7. Concentration (F5.3)

How well are you able to concentrate?

1 = Not at all | 2 = A little | 3 = A moderate amount | 4 = Very much | 5 = Extremely

8. Safety (F16.1)

How safe do you feel in your daily life?

1 = Not at all | 2 = A little | 3 = A moderate amount | 4 = Very much | 5 = Extremely

9. Physical environment (F22.1)

How healthy is your physical environment?

1 = Not at all | 2 = A little | 3 = A moderate amount | 4 = Very much | 5 = Extremely

10. Energy for daily life (F2.1)

Do you have enough energy for everyday life?

1 = Not at all | 2 = A little | 3 = Moderately | 4 = Mostly | 5 = Completely

11. Bodily appearance (F7.1)

Are you able to accept your bodily appearance?

1 = Not at all | 2 = A little | 3 = Moderately | 4 = Mostly | 5 = Completely

12. Financial resources (F18.1)

Have you enough money to meet your needs?

1 = Not at all | 2 = A little | 3 = Moderately | 4 = Mostly | 5 = Completely

13. Information availability (F20.1)

How available is the information that you need in your day-to-day life?

1 = Not at all | 2 = A little | 3 = Moderately | 4 = Mostly | 5 = Completely

14. Leisure opportunities (F21.1)

To what extent do you have the opportunity for leisure activities?

1 = Not at all | 2 = A little | 3 = Moderately | 4 = Mostly | 5 = Completely

15. Mobility (F9.1)

How well are you able to get around?

1 = Very poor | 2 = Poor | 3 = Neither poor nor good | 4 = Good | 5 = Very good

16. Satisfaction with sleep (F3.3)

How satisfied are you with your sleep?

1 = Very dissatisfied | 2 = Dissatisfied | 3 = Neither satisfied nor dissatisfied | 4 = Satisfied | 5 = Very satisfied

17. Daily activities (F10.3)

How satisfied are you with your ability to perform daily living activities?

1 = Very dissatisfied | 2 = Dissatisfied | 3 = Neither satisfied nor dissatisfied | 4 = Satisfied | 5 = Very satisfied

18. Capacity for work (F12.4)

How satisfied are you with your capacity for work?

1 = Very dissatisfied | 2 = Dissatisfied | 3 = Neither satisfied nor dissatisfied | 4 = Satisfied | 5 = Very satisfied

19. Self-satisfaction (F6.3)

How satisfied are you with yourself?

1 = Very dissatisfied | 2 = Dissatisfied | 3 = Neither satisfied nor dissatisfied | 4 = Satisfied | 5 = Very satisfied

20. Personal relationships (F13.3)

How satisfied are you with your personal relationships?

1 = Very dissatisfied | 2 = Dissatisfied | 3 = Neither satisfied nor dissatisfied | 4 = Satisfied | 5 = Very satisfied

21. Sex life (F15.3)

How satisfied are you with your sex life?

1 = Very dissatisfied | 2 = Dissatisfied | 3 = Neither satisfied nor dissatisfied | 4 = Satisfied | 5 = Very satisfied

22. Support from friends (F14.4)

How satisfied are you with the support you get from your friends?

1 = Very dissatisfied | 2 = Dissatisfied | 3 = Neither satisfied nor dissatisfied | 4 = Satisfied | 5 = Very satisfied

23. Living conditions (F17.3)

How satisfied are you with the conditions of your living place?

1 = Very dissatisfied | 2 = Dissatisfied | 3 = Neither satisfied nor dissatisfied | 4 = Satisfied | 5 = Very satisfied

24. Access to health services (F19.3)

How satisfied are you with your access to health services?

1 = Very dissatisfied | 2 = Dissatisfied | 3 = Neither satisfied nor dissatisfied | 4 = Satisfied | 5 = Very satisfied

25. Transport (F23.3)

How satisfied are you with your transport?

1 = Very dissatisfied | 2 = Dissatisfied | 3 = Neither satisfied nor dissatisfied | 4 = Satisfied | 5 = Very satisfied

26. Negative feelings (F8.1)

How often do you have negative feelings, such as blue mood, despair, anxiety, depression?

1 = Never | 2 = Seldom | 3 = Quite often | 4 = Very often | 5 = Always

DUREL – Duke University Religion Index (5 items)

Assesses three dimensions: Organizational Religiosity (1 item), Non-Organizational Religiosity (1 item), and Intrinsic Religiosity (3 items). Due to copyright restrictions, items are not reproduced.

Appendix C – Duke University Religion Index (DUREL)

English Version (5 items)

Original source: Koenig & Büssing (2010)

Instructions

The following questions ask about religious involvement. Please select the response that best represents your situation.

C1. Organizational Religiosity (Item 1)

1. How often do you attend church or other religious meetings?

- 1 — Never
- 2 — Once a year or less
- 3 — A few times a year
- 4 — A few times a month
- 5 — Once a week
- 6 — More than once a week

C2. Non-Organizational Religiosity (Item 2)

2. How often do you spend time in private religious activities such as prayer, meditation, or Bible study?

- 1 — Rarely or never
- 2 — Once in a while
- 3 — A few times a week
- 4 — Once a day
- 5 — More than once a day

C3. Intrinsic Religiosity (Items 3–5)

3. In my life, I experience the presence of the Divine (i.e., God).

4. My religious beliefs are what really lie behind my whole approach to life.

5. I try hard to carry my religion over into all other dealings in life.

- 1 — Definitely not true
- 2 — Tends not to be true
- 3 — Unsure

- 4 — Tends to be true
- 5 — Definitely true

CRE/RCOPE (87 items)

Composed of Positive Religious Coping (52 items) and Negative Religious Coping (35 items). The scale is copyrighted; therefore, only structural description is provided.

APPENDIX D – Religious Coping Inventory (87 items)

Each item is rated on a 5-point Likert scale as follows:
1 = Not at all
2 = A little
3 = A moderate amount
4 = Quite a bit
5 = Very much

D1. I prayed for the well-being of others

1 = Not at all
2 = A little
3 = A moderate amount
4 = Quite a bit
5 = Very much

D2. I sought God's love and protection

1 = Not at all
2 = A little
3 = A moderate amount
4 = Quite a bit
5 = Very much

D3. I asked God to help me forgive other people

1 = Not at all
2 = A little
3 = A moderate amount
4 = Quite a bit
5 = Very much

D4. I rebelled against God and His plans

1 = Not at all
2 = A little
3 = A moderate amount
4 = Quite a bit
5 = Very much

D5. I sought a stronger connection with God

1 = Not at all
2 = A little

3 = A moderate amount
4 = Quite a bit
5 = Very much

D6. I questioned God's love for me

1 = Not at all
2 = A little
3 = A moderate amount
4 = Quite a bit
5 = Very much

D7. I did not do much; I just waited for God to solve my problems

1 = Not at all
2 = A little
3 = A moderate amount
4 = Quite a bit
5 = Very much

D8. I looked for a religious house or place of prayer

1 = Not at all
2 = A little
3 = A moderate amount
4 = Quite a bit
5 = Very much

D9. I wondered whether evil had something to do with this situation

1 = Not at all
2 = A little
3 = A moderate amount
4 = Quite a bit
5 = Very much

D10. I tried to work for social well-being

1 = Not at all
2 = A little
3 = A moderate amount
4 = Quite a bit
5 = Very much

D11. I pleaded with God to make everything turn out well

1 = Not at all
2 = A little
3 = A moderate amount

4 = Quite a bit
5 = Very much

D12. I sought protection and guidance from spiritual entities (saints, spirits, orixás, etc.)

1 = Not at all
2 = A little
3 = A moderate amount
4 = Quite a bit
5 = Very much

D13. I sought strength, support, and guidance from God

1 = Not at all
2 = A little
3 = A moderate amount
4 = Quite a bit
5 = Very much

D14. I tried to join with others who shared my faith

1 = Not at all
2 = A little
3 = A moderate amount
4 = Quite a bit
5 = Very much

D15. I felt dissatisfied with the religious leaders of my institution

1 = Not at all
2 = A little
3 = A moderate amount
4 = Quite a bit
5 = Very much

D16. I read spiritual/religious teachings to understand and deal with the situation

1 = Not at all
2 = A little
3 = A moderate amount
4 = Quite a bit
5 = Very much

D17. I asked God to help me find a new purpose in life

1 = Not at all
2 = A little

3 = A moderate amount
4 = Quite a bit
5 = Very much

D18. I had difficulty receiving comfort from my religious beliefs

1 = Not at all
2 = A little
3 = A moderate amount
4 = Quite a bit
5 = Very much

D19. I sought love and care from members of my religious institution

1 = Not at all
2 = A little
3 = A moderate amount
4 = Quite a bit
5 = Very much

D20. I tried to stop thinking about my problems by thinking about God

1 = Not at all
2 = A little
3 = A moderate amount
4 = Quite a bit
5 = Very much

D21. I went to a religious temple

1 = Not at all
2 = A little
3 = A moderate amount
4 = Quite a bit
5 = Very much

D22. I did the best I could and surrendered the situation to God

1 = Not at all
2 = A little
3 = A moderate amount
4 = Quite a bit
5 = Very much

D23. I wondered whether God was punishing me for my lack of faith

1 = Not at all
2 = A little
3 = A moderate amount

4 = Quite a bit
5 = Very much

D24. I practiced moral and/or material acts of charity

1 = Not at all
2 = A little
3 = A moderate amount
4 = Quite a bit
5 = Very much

D25. I felt that God was acting together with me

1 = Not at all
2 = A little
3 = A moderate amount
4 = Quite a bit
5 = Very much

D26. I implored God to make things turn out well

1 = Not at all
2 = A little
3 = A moderate amount
4 = Quite a bit
5 = Very much

D27. I thought about spiritual matters to divert my attention from my problems

1 = Not at all
2 = A little
3 = A moderate amount
4 = Quite a bit
5 = Very much

D28. I understood why I was suffering through religion and tried to change my actions

1 = Not at all
2 = A little
3 = A moderate amount
4 = Quite a bit
5 = Very much

D29. I sought guidance from my higher spiritual guide (guardian angel, mentor, etc.)

1 = Not at all
2 = A little

3 = A moderate amount
4 = Quite a bit
5 = Very much

D30. I turned to God to find a new direction in life

1 = Not at all
2 = A little
3 = A moderate amount
4 = Quite a bit
5 = Very much

D31. I tried to provide spiritual comfort to other people

1 = Not at all
2 = A little
3 = A moderate amount
4 = Quite a bit
5 = Very much

D32. I wondered whether God had abandoned me

1 = Not at all
2 = A little
3 = A moderate amount
4 = Quite a bit
5 = Very much

D33. I asked God to help me be better and make fewer mistakes

1 = Not at all
2 = A little
3 = A moderate amount
4 = Quite a bit
5 = Very much

D34. I thought that what happened could bring me closer to God

1 = Not at all
2 = A little
3 = A moderate amount
4 = Quite a bit
5 = Very much

D35. I did not try to deal with the situation; I just waited for God to take my worries away

1 = Not at all
2 = A little
3 = A moderate amount

4 = Quite a bit
5 = Very much

D36. I felt that evil was trying to distance me from God

1 = Not at all
2 = A little
3 = A moderate amount
4 = Quite a bit
5 = Very much

D37. I surrendered the situation to God after doing everything I could

1 = Not at all
2 = A little
3 = A moderate amount
4 = Quite a bit
5 = Very much

D38. I prayed to discover the purpose of my life

1 = Not at all
2 = A little
3 = A moderate amount
4 = Quite a bit
5 = Very much

D39. I performed spiritual acts or rituals

1 = Not at all
2 = A little
3 = A moderate amount
4 = Quite a bit
5 = Very much

D40. I acted in collaboration with God to solve my problems

1 = Not at all
2 = A little
3 = A moderate amount
4 = Quite a bit
5 = Very much

D41. I wondered whether my religious institution had abandoned me

1 = Not at all
2 = A little
3 = A moderate amount
4 = Quite a bit
5 = Very much

D42. I focused my thoughts on religion to stop worrying about my problems

1 = Not at all
2 = A little
3 = A moderate amount
4 = Quite a bit
5 = Very much

D43. I sought a total spiritual reawakening

1 = Not at all
2 = A little
3 = A moderate amount
4 = Quite a bit
5 = Very much

D44. I sought spiritual support from leaders of my religious community

1 = Not at all
2 = A little
3 = A moderate amount
4 = Quite a bit
5 = Very much

D45. I prayed for a miracle

1 = Not at all
2 = A little
3 = A moderate amount
4 = Quite a bit
5 = Very much

D46. I followed spiritual advice aimed at improving my physical or psychological state

1 = Not at all
2 = A little
3 = A moderate amount
4 = Quite a bit
5 = Very much

D47. I trusted that God was with me

1 = Not at all
2 = A little
3 = A moderate amount
4 = Quite a bit
5 = Very much

D48. I sought spiritual help to overcome resentments and hurts

1 = Not at all
2 = A little
3 = A moderate amount
4 = Quite a bit
5 = Very much

D49. I sought God's mercy

1 = Not at all
2 = A little
3 = A moderate amount
4 = Quite a bit
5 = Very much

D50. I thought that God did not exist

1 = Not at all
2 = A little
3 = A moderate amount
4 = Quite a bit
5 = Very much

D51. I questioned whether even God has limits

1 = Not at all
2 = A little
3 = A moderate amount
4 = Quite a bit
5 = Very much

D52. I watched religious or spirituality-focused programs or films

1 = Not at all
2 = A little
3 = A moderate amount
4 = Quite a bit
5 = Very much

D53. I convinced myself that forces of evil acted to make all this happen

1 = Not at all
2 = A little
3 = A moderate amount
4 = Quite a bit
5 = Very much

D54. I sought help or comfort in religious literature

1 = Not at all
2 = A little
3 = A moderate amount
4 = Quite a bit
5 = Very much

D55. I offered spiritual support to my family and friends

1 = Not at all
2 = A little
3 = A moderate amount
4 = Quite a bit
5 = Very much

D56. I asked for forgiveness for my mistakes

1 = Not at all
2 = A little
3 = A moderate amount
4 = Quite a bit
5 = Very much

D57. I participated in spiritual healing sessions

1 = Not at all
2 = A little
3 = A moderate amount
4 = Quite a bit
5 = Very much

D58. I acted in partnership with God, collaborating with Him

1 = Not at all
2 = A little
3 = A moderate amount
4 = Quite a bit
5 = Very much

D59. I wondered whether God allowed this to happen because of my mistakes

1 = Not at all
2 = A little
3 = A moderate amount
4 = Quite a bit
5 = Very much

D60. I attended religious/spiritual services or sessions

1 = Not at all
2 = A little
3 = A moderate amount
4 = Quite a bit
5 = Very much

D61. I tried to do the best I could and let God do the rest

1 = Not at all
2 = A little
3 = A moderate amount
4 = Quite a bit
5 = Very much

D62. I voluntarily engaged in activities for the good of others

1 = Not at all
2 = A little
3 = A moderate amount
4 = Quite a bit
5 = Very much

D63. I listened to and/or sang religious songs

1 = Not at all
2 = A little
3 = A moderate amount
4 = Quite a bit
5 = Very much

D64. I knew I could not handle the situation, so I just waited for God to take control

1 = Not at all
2 = A little
3 = A moderate amount
4 = Quite a bit
5 = Very much

D65. I evaluated my actions, thoughts, and feelings, trying to improve them according to religious teachings

1 = Not at all
2 = A little
3 = A moderate amount
4 = Quite a bit
5 = Very much

D66. I received help through the laying on of hands

1 = Not at all
2 = A little
3 = A moderate amount
4 = Quite a bit
5 = Very much

D67. I sought help through meditation

1 = Not at all
2 = A little
3 = A moderate amount
4 = Quite a bit
5 = Very much

D68. I sought or engaged in spiritual treatments

1 = Not at all
2 = A little
3 = A moderate amount
4 = Quite a bit
5 = Very much

D69. I tried to deal with the situation on my own, without God's help

1 = Not at all
2 = A little
3 = A moderate amount
4 = Quite a bit
5 = Very much

D70. I tried to find a teaching from God in what happened

1 = Not at all
2 = A little
3 = A moderate amount
4 = Quite a bit
5 = Very much

D71. I tried to build a strong relationship with a higher power

1 = Not at all
2 = A little
3 = A moderate amount
4 = Quite a bit
5 = Very much

D72. I bought or subscribed to magazines about God and spiritual matters

1 = Not at all
2 = A little
3 = A moderate amount
4 = Quite a bit
5 = Very much

D73. I felt that my religious group seemed to be rejecting or ignoring me

1 = Not at all
2 = A little
3 = A moderate amount
4 = Quite a bit
5 = Very much

D74. I participated in religious or spiritual practices, activities, or celebrations

1 = Not at all
2 = A little
3 = A moderate amount
4 = Quite a bit
5 = Very much

D75. I set up a place of prayer in my home

1 = Not at all
2 = A little
3 = A moderate amount
4 = Quite a bit
5 = Very much

D76. I tried to deal with my feelings without asking for God's help

1 = Not at all
2 = A little
3 = A moderate amount
4 = Quite a bit
5 = Very much

D77. I sought guidance in sacred books

1 = Not at all
2 = A little
3 = A moderate amount
4 = Quite a bit
5 = Very much

D78. I wondered what I had done for God to punish me

1 = Not at all
2 = A little
3 = A moderate amount
4 = Quite a bit
5 = Very much

D79. I tried to change my life path and follow God's path

1 = Not at all
2 = A little
3 = A moderate amount
4 = Quite a bit
5 = Very much

D80. I tried to talk to my higher self

1 = Not at all
2 = A little
3 = A moderate amount
4 = Quite a bit
5 = Very much

D81. I turned to spirituality

1 = Not at all
2 = A little
3 = A moderate amount
4 = Quite a bit
5 = Very much

D82. I sought God's help to free myself from negative or harmful feelings

1 = Not at all
2 = A little
3 = A moderate amount
4 = Quite a bit
5 = Very much

D83. I blamed God for the situation, for allowing it to happen

1 = Not at all
2 = A little
3 = A moderate amount
4 = Quite a bit
5 = Very much

D84. I questioned whether God truly cared

1 = Not at all
2 = A little
3 = A moderate amount
4 = Quite a bit
5 = Very much

D85. I prayed individually and did what I felt was spiritually meaningful to me

1 = Not at all
2 = A little
3 = A moderate amount
4 = Quite a bit
5 = Very much

D86. I reflected on whether I was going against God's laws and tried to change my attitude

1 = Not at all
2 = A little
3 = A moderate amount
4 = Quite a bit
5 = Very much

D87. I sought a house of God

1 = Not at all
2 = A little
3 = A moderate amount
4 = Quite a bit
5 = Very much

BDI – Beck Depression Inventory-II (21 items)

Assesses depressive symptoms across cognitive, affective, and somatic dimensions. Full item text cannot be reproduced due to copyright.

APPENDIX F – Beck Depression Inventory (BDI)

Each item consists of four statements increasing in severity. Select the one that best describes how you have been feeling during the past week, including today.

F1.

0 I do not feel sad.

1 I feel sad.

2 I am sad all the time and I can't snap out of it.

3 I am so sad and unhappy that I can't stand it.

F2.

0 I am not particularly discouraged about the future.

1 I feel discouraged about the future.

2 I feel I have nothing to look forward to.

3 I feel the future is hopeless and that things cannot improve.

F3.

0 I do not feel like a failure.

1 I feel I have failed more than the average person.

2 As I look back on my life, all I can see is a lot of failures.

3 I feel I am a complete failure as a person.

F4.

0 I get as much satisfaction out of things as I used to.

1 I don't enjoy things the way I used to.

2 I don't get real satisfaction out of anything anymore.

3 I am dissatisfied or bored with everything.

F5.

0 I don't feel particularly guilty.

1 I feel guilty a good part of the time.

2 I feel quite guilty most of the time.

3 I feel guilty all of the time.

F6.

0 I don't feel I am being punished.

1 I feel I may be punished.

2 I expect to be punished.

3 I feel I am being punished.

F7.

0 I don't feel disappointed in myself.

1 I am disappointed in myself.

2 I am disgusted with myself.

3 I hate myself.

F8.

0 I don't feel I am any worse than anybody else.

1 I am critical of myself for my weaknesses or mistakes.

2 I blame myself all the time for my faults.

3 I blame myself for everything bad that happens.

F9.

0 I don't have any thoughts of killing myself.

1 I have thoughts of killing myself, but I would not carry them out.

2 I would like to kill myself.

3 I would kill myself if I had the chance.

F10.

0 I don't cry anymore than usual.

1 I cry more now than I used to.

2 I cry all the time now.

3 I used to be able to cry, but now I can't cry even though I want to.

F11.

0 I am no more irritated by things than I ever was.

1 I am slightly more irritated now than usual.

2 I am quite annoyed or irritated a good deal of the time.

3 I feel irritated all the time.

F12.

0 I have not lost interest in other people.

1 I am less interested in other people than I used to be.

2 I have lost most of my interest in other people.

3 I have lost all of my interest in other people.

F13.

0 I make decisions about as well as I ever could.

1 I put off making decisions more than I used to.

2 I have greater difficulty in making decisions more than I used to.

3 I can't make decisions at all anymore.

F14.

0 I don't feel that I look any worse than I used to.

1 I am worried that I am looking old or unattractive.

2 I feel there are permanent changes in my appearance that make me look unattractive.

3 I believe that I look ugly.

F15.

0 I can work about as well as before.

1 It takes an extra effort to get started at doing something.

2 I have to push myself very hard to do anything.

3 I can't do any work at all.

F16.

0 I can sleep as well as usual.

1 I don't sleep as well as I used to.

2 I wake up 1–2 hours earlier than usual and find it hard to get back to sleep.

3 I wake up several hours earlier and cannot get back to sleep.

F17.

0 I don't get more tired than usual.

1 I get tired more easily than I used to.

2 I get tired from doing almost anything.

3 I am too tired to do anything.

F18.

0 My appetite is no worse than usual.

1 My appetite is not as good as it used to be.

2 My appetite is much worse now.

3 I have no appetite at all anymore.

F19.

0 I haven't lost much weight, if any, lately.

1 I have lost more than five pounds.

2 I have lost more than ten pounds.

3 I have lost more than fifteen pounds.

F20.

0 I am no more worried about my health than usual.

1 I am worried about aches, pains, upset stomach, or constipation.

2 I am very worried about physical problems, and it's hard to think of anything else.

3 I am so worried about my physical problems that I cannot think of anything else.

F21.

0 I have not noticed any recent change in my interest in sex.

1 I am less interested in sex than I used to be.

2 I have almost no interest in sex.

3 I have lost interest in sex completely.

BAI – Beck Anxiety Inventory (21 items)

Assesses physical and cognitive symptoms of anxiety. Full item text is restricted by copyright.

APPENDIX E – Beck Anxiety Inventory (BAI)

Each item is rated on a 4-point Likert scale as follows:
0 = Not at all
1 = Mildly, but it didn't bother me much
2 = Moderately – it wasn't pleasant at times
3 = Severely – it bothered me a lot

E1. Numbness or tingling

0 = Not at all
1 = Mildly, but it didn't bother me much
2 = Moderately – it wasn't pleasant at times
3 = Severely – it bothered me a lot

E2. Feeling hot

0 = Not at all
1 = Mildly, but it didn't bother me much
2 = Moderately – it wasn't pleasant at times
3 = Severely – it bothered me a lot

E3. Wobbliness in legs

0 = Not at all
1 = Mildly, but it didn't bother me much
2 = Moderately – it wasn't pleasant at times
3 = Severely – it bothered me a lot

E4. Unable to relax

0 = Not at all
1 = Mildly, but it didn't bother me much
2 = Moderately – it wasn't pleasant at times
3 = Severely – it bothered me a lot

E5. Fear of worst happening

0 = Not at all
1 = Mildly, but it didn't bother me much
2 = Moderately – it wasn't pleasant at times
3 = Severely – it bothered me a lot

E6. Dizzy or lightheaded

0 = Not at all
1 = Mildly, but it didn’t bother me much
2 = Moderately – it wasn’t pleasant at times
3 = Severely – it bothered me a lot

E7. Heart pounding / racing

0 = Not at all
1 = Mildly, but it didn’t bother me much
2 = Moderately – it wasn’t pleasant at times
3 = Severely – it bothered me a lot

E8. Unsteady

0 = Not at all
1 = Mildly, but it didn’t bother me much
2 = Moderately – it wasn’t pleasant at times
3 = Severely – it bothered me a lot

E9. Terrified or afraid

0 = Not at all
1 = Mildly, but it didn’t bother me much
2 = Moderately – it wasn’t pleasant at times
3 = Severely – it bothered me a lot

E10. Nervous

0 = Not at all
1 = Mildly, but it didn’t bother me much
2 = Moderately – it wasn’t pleasant at times
3 = Severely – it bothered me a lot

E11. Feeling of choking

0 = Not at all
1 = Mildly, but it didn’t bother me much
2 = Moderately – it wasn’t pleasant at times
3 = Severely – it bothered me a lot

E12. Hands trembling

0 = Not at all
1 = Mildly, but it didn’t bother me much
2 = Moderately – it wasn’t pleasant at times
3 = Severely – it bothered me a lot

E13. Shaky / unsteady

0 = Not at all
1 = Mildly, but it didn’t bother me much
2 = Moderately – it wasn’t pleasant at times
3 = Severely – it bothered me a lot

E14. Fear of losing control

0 = Not at all
1 = Mildly, but it didn’t bother me much
2 = Moderately – it wasn’t pleasant at times
3 = Severely – it bothered me a lot

E15. Difficulty in breathing

0 = Not at all
1 = Mildly, but it didn’t bother me much
2 = Moderately – it wasn’t pleasant at times
3 = Severely – it bothered me a lot

E16. Fear of dying

0 = Not at all
1 = Mildly, but it didn’t bother me much
2 = Moderately – it wasn’t pleasant at times
3 = Severely – it bothered me a lot

E17. Scared

0 = Not at all
1 = Mildly, but it didn’t bother me much
2 = Moderately – it wasn’t pleasant at times
3 = Severely – it bothered me a lot

E18. Indigestion

0 = Not at all
1 = Mildly, but it didn’t bother me much
2 = Moderately – it wasn’t pleasant at times
3 = Severely – it bothered me a lot

E19. Faint / lightheaded

0 = Not at all
1 = Mildly, but it didn’t bother me much
2 = Moderately – it wasn’t pleasant at times
3 = Severely – it bothered me a lot

E20. Face flushed

0 = Not at all

1 = Mildly, but it didn't bother me much

2 = Moderately – it wasn't pleasant at times

3 = Severely – it bothered me a lot

E21. Hot / cold sweats

0 = Not at all

1 = Mildly, but it didn't bother me much

2 = Moderately – it wasn't pleasant at times

3 = Severely – it bothered me a lot
